# General Board of Health

**Published:** 1853-11

**Authors:** 


### BIBLIOGRAPHICAL NOTICES.

---

*General Board of Health. Second Report on Quarantine. Yellow Fever. With appendices, presented in both Houses of Parliament, by command of her Majesty.* London, 1852. 8vo. pp. 414.

This is the second of a series of reports, of great practical value in a sanitary and commercial point of view; the first of which appeared in 1849, addressed to Parliament, on Quarantine and Cholera. We are advised that the third Report will contain the decisions of the International Sanitary Conference which has just terminated its labors at Paris.

The names of Edwin Chadwick, Esq., and Dr. J. Southwood Smith, appended to these reports, two of the committee of investigation, appointed by the Board of Health, are a sufficient guarantee of the manner in which the commission has been executed; while the subjects discussed particularly in this second report, now before us, are of a character that will secure for it a careful reading, wherever it can be had.

Indeed it is a source of regret, that this deeply interesting document had not been issued in a form that would insure for it a more general circulation, especially in those countries where Yellow Fever prevails endemically.

This second report presents to the reader several points of absorbing interest. While it purports to be an investigation into the necessity or otherwise of Quarantine laws, it takes hold of and discusses in a masterly manner the subject of Yellow Fever, contributing much towards the solution of several questions, hitherto involved in doubt and obscurity.

The inquiry professes to settle the leading points at issue, viz., the non-contagiousness of Yellow Fever, its domestic origin, and its similarity in type to miasmatic fevers.

The conclusions to which the General Board of Health have arrived, as set forth in this report, after a close and scientific investigation of a large amount of evidence, are presented under fifteen distinct heads, which may be summed up as follows:

That Yellow Fever epidemics may appear in different towns and remote parts of the same town, under circumstances in which communication with infected persons is impossible; that they are usually preceded by sporadic cases, which cases are common when no epidemic prevails; that they do not spread by any rule of gradual progression; that, when the fever is prevalent in a locality, rigid seclusion affords no security; that the entire removal from an infected district, often arrests the progress of the epidemic; that, the disease cannot be transmitted from one person to another in a hospital; that it is local or endemic in its origin, and those causes which influence its location are removeable, and in proportion as they are removed so will the fever subside, or appear in a milder form; that non-acclimatization is a predisposing cause for the disease; that there is no evidence to prove that Yellow Fever has ever been imported; and lastly, that "the means of protection from Yellow fever are not quarantine restrictions and sanitary cordons, but sanitary works and operations, having for their object the removal and prevention of the several localizing conditions, and when such permanent works are impracticable, the temporary removal, as far as may be possible, of the population from the infected localities."

The question, as to the necessity of the present rigid system of quarantine, as a preventive for the introduction of disease from abroad, is taken up and thoroughly investigated. The result of this inquiry is, that they cannot afford any real protection, and "that the substitution of Sanitary and Hygiene measures, for quarantine isolation and restriction, would afford more certain and effectual protection."

While we agree in most particulars with the opinions advanced by the General Board of Health on this subject, and entertain the most reliable confidence in the adoption of a rigid police system, for the prevention and spread of disease, and while we are decidedly in favor of such an entire modification of our quarantine laws, as will forever remove those arbitrary and oppressive features, which, in this enlightened age, ought to be looked upon by our law makers as alike cruel, unjust and injurious to commerce, inflicting unnecessary inconvenience on passengers and seamen, "while they maintain a false security in relation to the means of preventing disease"; we are not altogether prepared to sanction the propriety of admitting vessels into port that have come from unhealthy places, or that have had infectious disease on board during the voyage, until they have undergone the process of ventilation and purification beyond the limits of the Port.

In regard to the introduction of small pox, however, which forms an exception to the system proposed in this report, we should deem it advisable not only to detain the vessel for purification, but also the crew and passengers, or such of them as are laboring under the disease, until they have recovered.

In the form of an appendix there is an interesting report on Yellow Fever; by J. Gillkrest, M. D., Inspector General of Army Hospitals. The reply of Judge Howell to Sir George Murray, giving the reasons for his opinion (as one of the members of the Board of Inquiry into the origin of the Yellow Fever epidemic of 1828 in that Garrison,) that said epidemic had its origin in Gibralter. Remarks on the Yellow Fever of the West Indies and coast of Africa, by Dr. A. Browne, founded on statistical reports of the army and navy, &c., and a notice of the proceedings of a board of Inquiry held at the office of the army medical department 1849-50, on the nature of Yellow Fever. All of which are interesting and replete with valuable statistical and historical information on the subject of Yellow Fever.

Two beautiful engravings of Gibraltar accompany the book, as well as the Abstract of a Report, by James Wynne, M. D., on Epidemic Cholera as it prevailed in the United States in 1849 and '50—an American work of much merit on the etiology of that fatal scourge to the world.

